# TOWARDS A NOVEL MODULAR ARCHITECTURE FOR CERN RADIATION MONITORING

**DOI:** 10.1093/rpd/ncw308

**Published:** 2016-11-30

**Authors:** Hamza Boukabache, Michel Pangallo, Gael Ducos, Nicola Cardines, Antonio Bellotta, Ciarán Toner, Daniel Perrin, Doris Forkel-Wirth

**Affiliations:** 1 CERN, 385 route de Meyrin, CH-1211 Geneva 23, Switzerland

## Abstract

The European Organization for Nuclear Research (CERN) has the legal obligation to protect the public and the people working on its premises from any unjustified exposure to ionising radiation. In this context, radiation monitoring is one of the main concerns of the Radiation Protection Group. After 30 y of reliable service, the ARea CONtroller (ARCON) system is approaching the end of its lifecycle, which raises the need for new, more efficient radiation monitors with a high level of modularity to ensure better maintainability. Based on these two main principles, new detectors are currently being developed that will be capable of measuring very low dose rates down to 50 nSv h^−1^, whilst being able to measure radiation over an extensive range of 8 decades without any auto scaling. To reach these performances, CERN Radiation MOnitoring Electronics (CROME), the new generation of CERN radiation monitors, is based on the versatile architecture that includes new read-out electronics developed by the Instrumentation and Logistics section of the CERN Radiation Protection Group as well as a reconfigurable system on chip capable of performing complex processing calculations. Beside the capabilities of CROME to continuously measure the ambient dose rate, the system generates radiation alarms, provides interlock signals, drives alarm display units through a fieldbus and provides long-term, permanent and reliable data logging. The measurement tests performed during the first phase of the development show very promising results that pave the way to the second phase: the certification.

## INTRODUCTION

The European Organization for Nuclear Research (CERN) designs, develops and operates particle accelerators and different experiments for scientific research to understand the fundamental laws of the universe. These activities may generate stray radiation due to the interaction between beams and matter. The radiation detectors installed at several locations close to the beam lines and targets of these areas allow CERN to precisely monitor radiation levels. In contrast to the existing solutions for radiation monitoring^(1)^, the CERN Radiation MOnitoring Electronics (CROME) project makes use of all the CERN's in-house expertise to build a complete modular, versatile and reconfigurable technology. In addition to the innovation, this architecture will provide excellent service quality with considerable performance improvement for future developments in terms of both measurement level and scalability.

## NEEDS

**Figure 1.**
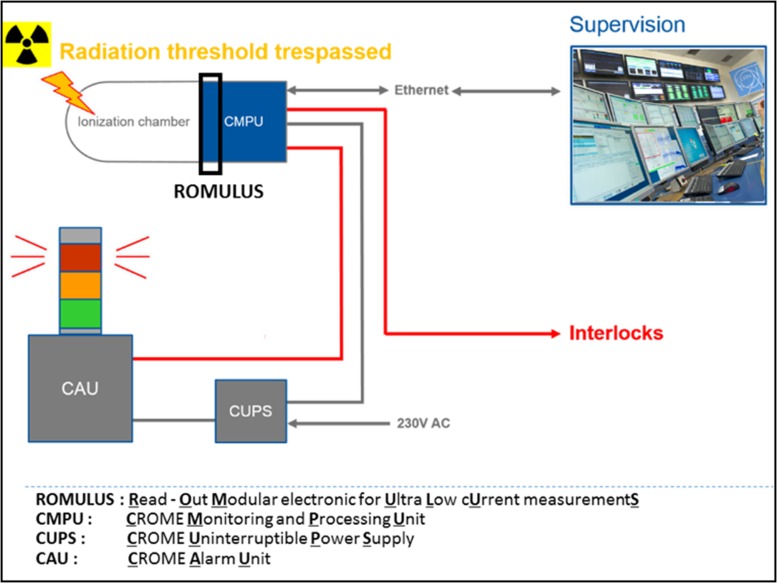
CROME's system architecture.

For the reasons outlined above, new monitors are currently being developed within the Radiation Instrumentation and Logistics section in the framework of ‘CROME project’. Included in the scope of the project may cite:

- Development of novel read-out electronics that will be capable of measuring very low dose rates at levels beyond the present state of the art whilst being able to measure radiation over an extensive range without any auto scaling. The continuous measurement is streamed towards a central software supervision system.
- As radiation protection equipment, the system shall be capable to trigger visual and audible alarms if the monitored dose rate exceeds a preset threshold (Cf. Figure 1). Furthermore, the system shall be capable to interlock machinery in case of high radiation levels (i.e. accelerators, RF cavities…) and systems that prevent personnel exposure to radiation (i.e. access systems).
- Replace in a first step 180 ARCON monitors^(1)^ and >100 Alarm units installed all over the CERN sites.

## SYSTEM ARCHITECTURE

The CROME project is one of the projects of the RAMSES program and aims at developing high performance, cost effective, low maintenance radiation monitors for CERN. The project encompasses the design, the conception, the development and the series production of a new generation of read-out electronics for radiation protection monitors (CROME Measuring and Processing Unit, CMPU) as well as alarm units (CROME Alarm Unit, CAU) and uninterruptible power supply (CROME Uninterruptible Power Supply, CUPS) as illustrated in Figure 1.

### CROME Measuring and Processing Unit

The CMPU is the main subsystem of CROME. The CMPU is composed in its basic configuration by:

- An ionisation chamber as the physical detector.
- A cylindrical metallic enclosure for the electronics that guarantees a protection index of IP54 (protection against dust and splashing water) and allows the electronics to be fixed to the ionisation chamber. The electronics part is composed of:
  - The read-out electronics.
  - The control and processing system.

### The read-out electronics

The read-out electronics is one of the major cores of the CMPU. It is completely modular, scalable and versatile through the assembly of elementary blocks that provide the following:

- An ultra-low current measurement down to femtoampere to ensure a continuous real-time measurement of ambient dose equivalent rates.
- A wide dynamic analysis of captured charges to ensure the acquisition of pulsed and transient radiation.
- A continuous tracking of some environmental parameters such as the temperature and the humidity.
- A high-voltage generation to polarise the detector.

Tables 1 and 2 below summarise the key performance requirements of the measurement part^(2, 3)^. One of the main challenges of the electronics is therefore its capability to ensure a very precise measurement while guaranteeing a wide spectrum bandwidth without the induction of any instability phenomenon. This trade-off is guaranteed by a number of feedbacks that ensure the control of the electronics.

**Table 1.** Front-end key performance requirements in current/charges.

Performance in term of	Range/value	Description
Input current range for continuous currents	From 2 fA to 250 nA	Within this range the specified accuracy requirements are fulfilled
Input current range for pulsed currents	≤500 nC per pulse	
Accuracy	±1%	Accuracy of the current measurement circuit within the input current range and within the limits of the external influence parameters
Maximum resolution	1 fA	Maximum resolution of the current measurement circuit defined for a measuring time of 60 s
Operational temperature range	from −15°C to 55°C	Within this range, the specified accuracy requirements are fulfilled

**Table 2.** Key performance requirements in equivalent ambient dose rate.

Detector	Measurement range	Conversion factor [A Sv^−1^ h^−1^]	Current range generated by the chamber
Argon-filled chamber	50 nSv h^−1^–0.1 Sv h^−1^	*γ*: 1.6 × 10^−6^	80 fA–160 nA
Hydrogen-filled chamber	50 nSv h^−1^–0.1 Sv h^−1^	*γ*: 1.13 × 10^−7^	5.7 fA–11.3 nA
		*n*: 4.0 × 10^−8^	2 fA–4 nA

The main advantages of the modular design are the following:

- Ability to upgrade the various boards individually.
- Maintainability and cost reduction: replace only the faulty board when it is needed.
- Possibility of decoupling the sensitive analogue front-end from the rest of the system (e.g. placing the high voltage and power supply functions, usually sources of heat and electromagnetic interference, far away from the sensitive analogue front-end).

**Figure 2.**
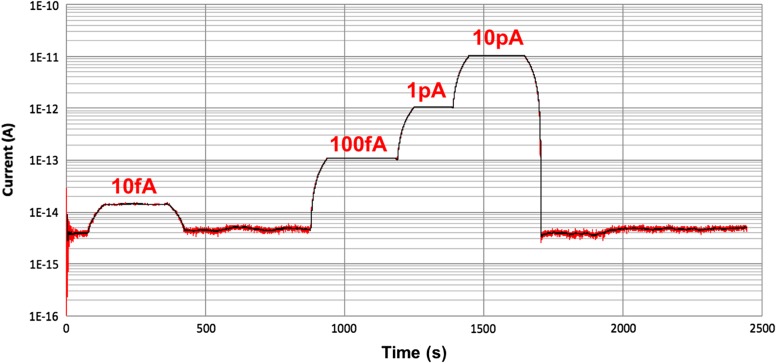
CROME's analogue front-end tests: dynamic characterisation over the first 4 decades.

**Figure 3.**
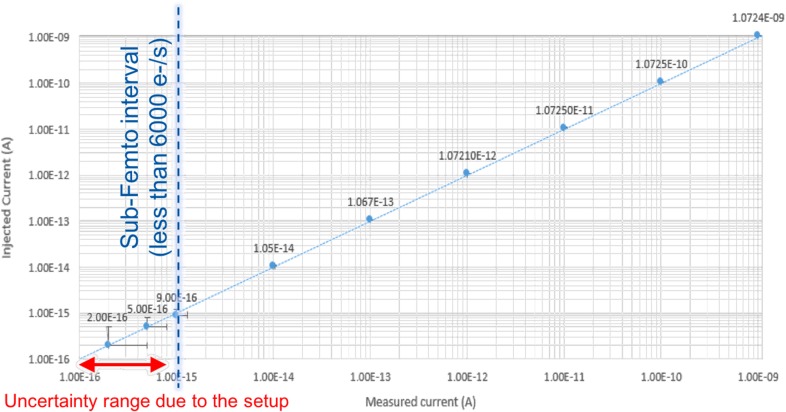
CROME's analogue front-end tests: linearity characterisation over 7 decades.

 The first characterisations of the analogue front-end electronics have been performed by a certified laboratory in order to validate the measurement principle for very low currents. As shown in Figure 2, the system is capable to dynamically measure different levels of currents. By repeating the tests, it is demonstrated that the system is capable to cover at least seven orders of magnitude ranging from the femtoampere to nanoamperes. The system has shown a very good linearity (Cf. Figure 3) despite the systematic relative error due to both a parasitic gain and a constant offset.

**Figure 4.**
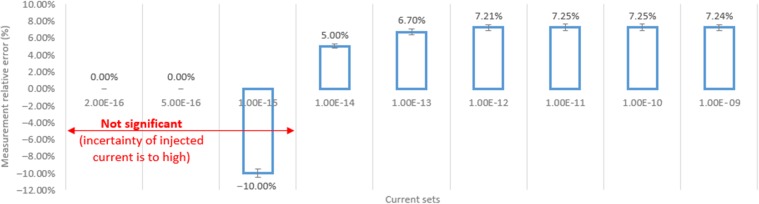
CROME's analogue front-end tests: relative error of the measurement.

Therefore, as shown in Figure 4, for a non-calibrated system, the relative error is equal above the femtoampere range to 7.2% and corresponds as shown in Figure 3 to a gain error of 7.2%. Indeed, for an injected current of 1 pA the system measures 1.072 pA and for an injected current of 1 nA the system measures 1072 nA.

Unfortunately, under the femtoampere level, the calibrated test bench has a too significant uncertainty that makes the interpretation of the results inaccurate. This uncertainty at the level of hundreds of attoampere is in the order of some 300 aA.

Based on the preliminary results shown in Figure 3 and based on the conversion factors summarised in Table 2, the system would meet the requirements in term of measured dose. In other words, the designed read-out electronics covers the required measurement range:

- For neutron radiation, using a hydrogen detector: [2 fA–4 nA] → [50 nSv h^−^^1^–0.1 Sv h^−1^].
- For gamma radiation, using a hydrogen detector: [5.7 fA–11.3 nA] → [50 nSv h^−1^–0.1 Sv h^−1^].
- For gamma radiation, using an argon detector: [80 fA–160nA] → [50 nSv h^−1^–0.1 Sv h^−1^].

### The control and processing system

The aim of the embedded digital control and processing system is to collect and process data from various sensors (e.g. analogue to digital converters, digital to analogue converters, temperature and humidity), to send commands to the Alarm Units and to send measurement results to a central supervisory system *via* the CERN internal technical TCP/IP communication network. The embedded digital system also integrates a wide range of auto-diagnostics in order to make sure that the device is working properly. It is also able to perform auto-calibration functions based on experimental models to ensure that the best measurements are taken in a wide range of environmental conditions. All the real-time data acquired and processed are stored in a mass storage media, ensuring a reliable long-term logging of all the information treated by the digital control and processing system.

The digital control and processing system of CROME is also modular. It is based on a System on Chip (SoC) that integrates a dual core ARM Cortex-A9 microprocessor in the same die of a Field-Programmable Gate Array (FPGA) programmable logic, granting both great flexibility and processing power to the digital system. The FPGA part of the SoC^(4)^ is commonly referred as Programmable Logic, while the dual ARM Cortex-A9 is referred as the Processing System. The first part is used for low level or low latency functionalities:

- Communication with digitalisation circuits and fast commutation chips.
- Instrumentation of environmental sensors such as temperatures or humidity.
- Generation of interlock signals.
- Triggering of alarms.

 The FPGA ensures the extremely short response time functions, unobtainable with a simple microcontroller. The processing part integrates high-level functions, manages the TCP/IP communication and the logging of data on a mass media storage. It runs a reliable and fully customisable distribution of Linux for embedded system. Parameters and data are exchanged between the processing system and programmable logic using a dedicated AXI bus.

### CROME Alarm Unit

The CAU provides visual and audible alerts to people when CROME monitor detects radiation above defined thresholds. Three coloured lamps and one siren are triggered according to three defined alert modes:

- System OK.
- Alert (first alert threshold).
- Alarm (evacuation).

 Because of the criticality of this safety device, the reliability requirement is very high. The design has been made in order to reach a Safety Integrity Level 2 according to the IEC61508 standard. In order to reach this high functional reliability, the CAU embeds all necessary self-diagnostic tests to guarantee the availability of the alarm function:

- Auto-diagnostic of lights and sound devices.
- Permanent power tests.
- Intelligent fieldbus interface with transmission consistency diagnostic.

### CROME Uninterruptible Power Supply

CUPS stands for CROME Uninterruptible Power Supply. This unit provides a constant 24V DC power supply to CROME units such as the CMPU radiation detector and the CAU. In normal conditions, the DC power is generated from the 230 V AC main power network. In case of a main power failure, the DC power output is maintained through the internal battery. A permanent diagnostic of this battery ensures the availability of the backup power at any time.

## CONCLUSION

This paper shows very preliminary results of the tests performed in a certified metrological laboratory for the measurement part. A certified COFRAC (French Accreditation Committee recognised by European Cooperation for Accreditation) laboratory has accredited the results presented. However, there are still many challenging issues to overcome. One of the major remaining studies is to assess the reliability of the design. Moreover, the front-end has to be to characterised below the femtoampere. This threshold corresponds to ≈ 6000 electrons per second and it is very difficult to find certified test benches capable to generate such low levels of current with high certainty and high accuracy.

## References

[1] Pangallo M., Boukabache H. and Perrin D. 2015 Study and development of a multiplexed radiation instrument solution for CERN facilities. IEEE International Symposium on Systems Engineering (ISSE). Rome, Italy. DOI: 10.1109/SysEng.2015.7302738.

[2] Theis C. et al Characterisation of ionisation chambers for a mixed radiation field and investigation of their suitability as radiation monitors for the LHC. Radiat. Prot. Dosim. 116(1–4 Pt 2), 170–174 (2005). 10.1093/rpd/nci097 16604621

[3] Mayer S. et al Response of neutron detectors to high-energy mixed radiation fields. Radiat. Prot. Dosim. 125(1–4), 289–292 (2007). 10.1093/rpd/ncm182 17337743

[4] Boukabache H. et al System-on-Chip integration of a new electromechanical impedance calculation method for aircraft structure health monitoring. Sensors 12(10), 13617–13635 (2012) doi:10.3390/s121013617. 23202013 10.3390/s121013617 PMC3545584

[5] Forkel-Wirth D. et al *Radiation monitoring system for the environment and safety project* Presented at the 5th ST Workshop, Echenevex, France, 28–30 January 2002.

